# Soil Moisture Retrieval from the Chinese GF-3 Satellite and Optical Data over Agricultural Fields

**DOI:** 10.3390/s18082675

**Published:** 2018-08-14

**Authors:** Linlin Zhang, Qingyan Meng, Shun Yao, Qiao Wang, Jiangyuan Zeng, Shaohua Zhao, Jianwei Ma

**Affiliations:** 1 Institute of Remote Sensing and Digital Earth, Chinese Academy of Sciences, Beijing 100101, China; zhangll@radi.ac.cn (L.Z.); zengjy@radi.ac.cn (J.Z.); 2 University of Chinese Academy of Sciences, Beijing 100049, China; 3 Sanya Institute of Remote Sensing, Sanya 572029, China; 4 DFH Satellite Co., Ltd., Beijing 100094, China; sugeryao@163.com; 5 Satellite Environment Center, Ministry of Environmental Protection, Beijing 100094, China; wangqiao@mep.gov.cn (Q.W.); zshyytt@126.com (S.Z.); 6 China Institute of Water Resources and Hydropower Research, Beijing 100038, China; majw@iwhr.com

**Keywords:** GF-3 satellite, soil moisture, simulation database, water cloud model

## Abstract

Timely and accurate soil moisture information is of great importance in agricultural monitoring. The Gaofen-3 (GF-3) satellite, the first C-band multi-polarization synthetic-aperture radar (SAR) satellite in China, provides valuable data sources for soil moisture monitoring. In this study, a soil moisture retrieval algorithm was developed for the GF-3 satellite based on a backscattering coefficient simulation database. We adopted eight optical vegetation indices to determine the relationships between these indices and vegetation water content (VWC) by combining Landsat-8 data and field measurements. A backscattering coefficient database was built using an advanced integral equation model (AIEM). The effects of vegetation on backscattering coefficients were corrected using the water cloud model (WCM) to obtain the bare soil backscattering coefficient ($\sigma_{soil}^{{}^{\circ}}$). Then, soil moisture retrievals were obtained at HH, VV and HH+VV combination respectively by minimizing the observed bare soil backscattering coefficient ($\sigma_{soil}^{{}^{\circ}}$) and the AIEM-simulated backscattering coefficient ($\sigma_{\text{soil-simu}}^{{}^{\circ}}$). Finally, the proposed algorithm was validated in agriculture region of wheat and corn in China using ground soil moisture measurements. The results showed that the normalized difference infrared index (NDII) had the best fit with measured VWC values (*R* = 0.885) among the eight vegetation water indices; thus, it was adopted to correct the effects of vegetation. The proposed algorithm using GF-3 satellite data performed well in soil moisture retrieval, and the scheme combining HH and VV polarization exhibited the highest accuracy, with a root mean square error (RMSE) of 0.044 m^3^m^−3^, followed by HH polarization (RMSE = 0.049 m^3^m^−3^) and VV polarization (RMSE = 0.053 m^3^m^−3^). Therefore, the proposed algorithm has good potential to operationally estimate soil moisture from the new GF-3 satellite data.

## 1. Introduction

Surface soil moisture plays a key role in hydrologic, agronomic, and meteorological process, and controls evaporation and transpiration fluxes from bare soil and vegetated areas, respectively [1,2,3,4]. In-situ measurements can provide highly accurate data on soil moisture; however, they are representative only over a very small spatial scale due to the strong heterogeneity of the land surface (e.g., vegetation, topography, and soil texture) [5]. Over the past few decades, microwave remote sensing, both active and passive, has demonstrated a great potential to provide surface soil moisture data at large scales in both time and space due to its high sensitivity to soil permittivity and because it provides all-time and all-weather coverage. The Soil Moisture Active Passive (SMAP) satellite mission developed by NASA was designed to make global mapping of high-resolution soil moisture using an L-band (active) radar and an L-band (passive) radiometer. The radiometer-only soil moisture product became the only soil moisture product for SMAP due to the hardware failure of the radar [6]. Passive microwave remote sensing can observe soil moisture with higher temporal resolution (e.g., 1~3 days) [7] than active radar especially the synthetic aperture radar (SAR) (typically the temporal resolution of a single SAR system is several weeks). However, passive radiometers often limits on spatial resolution (e.g., >25 km) while SAR can provide observations with much higher spatial resolution than the passive radiometers that can be used for many practical applications such as agricultural productivity estimation at a local scale [8].

Soil moisture retrieval from microwave SAR data generally employs both statistical (e.g., Oh [9] and Dubois models [10]) and physical models (e.g., the integral equation model (IEM) [11] and its update, advanced IEM (AIEM) [12]), which determine the complex relationships between surface parameters (e.g., soil moisture and surface roughness) and radar backscatter. Statistical models are developed based on the matching of large experimental datasets to radar measurements (frequency, polarization, and incidence angle) and geophysical variables (soil moisture and surface roughness). Although statistical models often work well on the same or similar datasets, they are generally site- dependent and thus limited, to a certain extent, in their application to other regions or datasets [13]. In contrast to statistical models, physical models account for the physics of the interactions between electromagnetic fields and natural surfaces, allowing the simulation of radar measurements over a wide range of geometric parameters and ground surface conditions [13,14].

Studies that have used radar data to estimate bare soil moisture have achieved promising results, with an accuracy of 3–6 vol % (e.g., [15,16,17,18,19]). However, the estimation of soil moisture in the presence of vegetation remains challenging due to the multiple-scattering effects of vegetation. Vegetation canopies have their own moisture, which scatters and attenuates electromagnetic radiation, which makes it difficult to discriminate the radar return due exclusively to soil moisture [20]. Therefore, a key problem is isolating the contribution of vegetation from the total radar backscatter. Physical models generally assume that the vegetation canopy contains a random distribution of scatter [21,22,23]. However, direct inversion of soil moisture from physical models of vegetation is generally not feasible because these models often involve a number of input parameters. By contrast, a semi-empirical forward model, e.g., a water cloud model (WCM), generally assumes that the vegetation canopy is a uniform layer of cloud water droplets, and the contribution of higher- order scattering is neglected [24]. Due to the simplicity and effectiveness of WCM, it has been widely used in both direct and inverse modeling of the scattering vegetated areas [25,26,27,28]. In the WCM, contribution of vegetation to the reflected radar signal is mainly characterized by vegetation optical depth. To minimize the effects of vegetation, optical vegetation indices (e.g., the normalized difference vegetation index, NDVI) are typically adopted as good proxies for vegetation optical depth [29,30,31]. Many studies have demonstrated that the accuracy of soil moisture estimates can be significantly improved when optical and SAR data are used jointly [32,33,34]. Therefore, soil moisture retrieval with combined use of SAR and optical data (particularly the optimal optical vegetation index) under vegetated areas is among the objectives of the present study.

Several soil moisture retrieval algorithms have been developed and tested for multiple SAR satellites operated at the L/C/X-bands, such as ALOS-2, Radarsat-2, Sentinel-1, and TerraSAR-X. The GaoFen-3 (GF-3) satellite, which is the first C-band radar satellite with quad-polarization and multi-angle capability for the civilian field in China, was launched on August 10, 2016 [35]. The GF-3 satellite has 12 imaging modes with an image resolution range of 1–500 m and image width range of 10–650 km. To the best of our knowledge, no study has investigated the capability of GF-3 in estimating soil moisture. Furthermore, soil moisture retrieval research by matching the simulated bare soil backscatter and observed bare soil backscatter at HH, VV and HH+VV three ways few has been done. Therefore, in the current study, we propose a soil moisture retrieval algorithm over agriculture fields using the GF-3 satellite and remotely sensed optical data with a backscattering coefficient simulation database. Ground soil moisture measurements in agriculture region with wheat and corn as the dominant crop type were used to assess the performance of the GF-3 satellite data in soil moisture estimation using the proposed algorithm.

The remainder of this paper is structured as follows: Section 2 introduces the study area. Section 3 provides a review of the datasets used. Section 4 describes the study methodology. Section 5 presents the results and discussion. Finally, our conclusions are provided in Section 6.

## 2. Study Area

**Figure 1:**
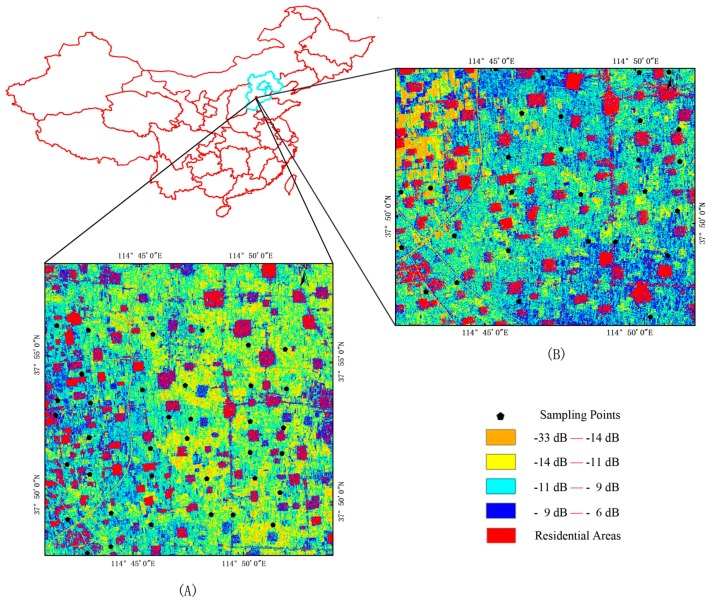
The geo-location of the Luancheng study area in China. The remote sensing images show the HH-polarized backscattering coefficients of GF-3 quad-polarization strip I (QPSI) data; (**A**) was acquired on 27 May 2017 with the crop type of wheat and (**B**) was acquired on 12 July 2017 with the crop type of corn. Red patches represent residential areas and black pentagons represent field measurement sampling points.

The Luancheng district of China (Figure 1) was selected as the study area to investigate the performance of the proposed soil moisture retrieval algorithm. It is located on the North China Plain (centered at 114.65° E, 37.88° N), covering approximately 25 km × 25 km (several orders of magnitude greater than the spatial resolution of the sensors used), which makes this area very suitable for testing the capability of SAR data in monitoring soil moisture. 

The climate in Luancheng is a typical sub-humid north-temperate, continental monsoon climate characterized by hot rainy summers, cold dry winters, and a short spring and autumn. Luancheng is also characterized by mainly cinnamon soil with high nutrient content, which is suitable for crop growth. The main grain crops planted in the study area are wheat and corn. Although the study area is not large, it has the representative characteristics of crop distribution on the North China Plain. To develop and validate the soil moisture retrieval algorithm developed for the GF-3 satellite, we selected 38 fields with a large variety of wheat and 28 fields with a large variety of corn to conduct ground measurements of soil and vegetation characteristics in 26–28 May 2017 and 12–17 July 2017.

## 3. Data and Preprocessing

### 3.1. In-Situ Field Measurements

In-situ field measurements of soil and vegetation parameters were conducted in 26–28 May 2017 for the wheat and 12–17 July 2017 for the corn, which is coincident with GF-3 and Landsat 8 satellite overpasses. 38 fields for wheat and 28 fields for corn (each field is an equilateral triangle with a length of 150 m) were selected to measure the parameters. Soil surface roughness was measured with a 1-m profile of a pin meter. For each field, roughness was processed by averaging the roughness parameters (root mean square height [*s*] and correlation length [*l*]) obtained from three sparsely distributed measurement points. Soil moisture was measured using an aluminium specimen box by the oven-drying method at a depth of 0–5 cm. Measurements were collected at three locations in each field, and average value was adopted. The water content of wheat and corn samples collected from randomly selected 1.0 m × 1.0 m squares was determined by weighing them before and after oven drying (wet weight − dry weight).

### 3.2. Satellite Data and Preprocessing

#### 3.2.1. GF-3 Satellite

GF-3 is the first Chinese civil C-band SAR. It was launched by the China Academy of Space Technology (CAST) on 10 August 2016 [36], into a polar Sun-synchronous orbit 755 km in altitude with a 26-day repeat cycle. The antenna is a wave-guide slot phased array with an area of 15 m × 15 m [37]. The GF-3 satellite has 12 imaging modes with a fine spatial resolution up to 1 m. 

During the field campaign, two GF-3 satellite images (27 May 2017 and 12 July 2017) of quad-polarization strip I (QPSI) mode downloaded from the website of China Centre For Resources Satellite Data and Application were obtained and processed up to level-1A single-look complex imagery (SLC). The main technical specifications of this imaging mode are listed in Table 1. The incidence angles of the two used GF-3 satellite image are 24° and 49°, respectively. Operating frequency is 5.4 GHz and the spatial resolution is 8 m. After image preprocessing, including calibration, filtering, and geo-coding, the backscattering coefficients of each polarization image were obtained. We focused only on co-polarizations (HH and VV). Because the backscattering coefficients of residential houses are higher than those of farmland area due to dihedral scattering [38], a threshold value was used to mask houses (red patches, Figure 1) to improve the clarity of the soil moisture map.

#### 3.2.2. Landsat-8 Operational Land Imager

The Landsat 8 satellite carries a two-sensor payload, including an operational land imager (OLI) and thermal infrared sensor (TIRS), which can acquire global moderate-resolution measurements of the Earth’s terrestrial and polar regions in the visible, near-infrared, short wave, and thermal infrared spectra [39]. The Landsat-8 images used in this study were downloaded from the United States Geological Survey (USGS) data archive (https://earthexplorer.usgs.gov/). We used the land surface reflectance product (Landsat Collection 1 Level-2, spatial resolution is 30 m), which has already been preprocessed, including radiation calibration and atmospheric correction. 

## 4. Methodology

**Figure 2:**
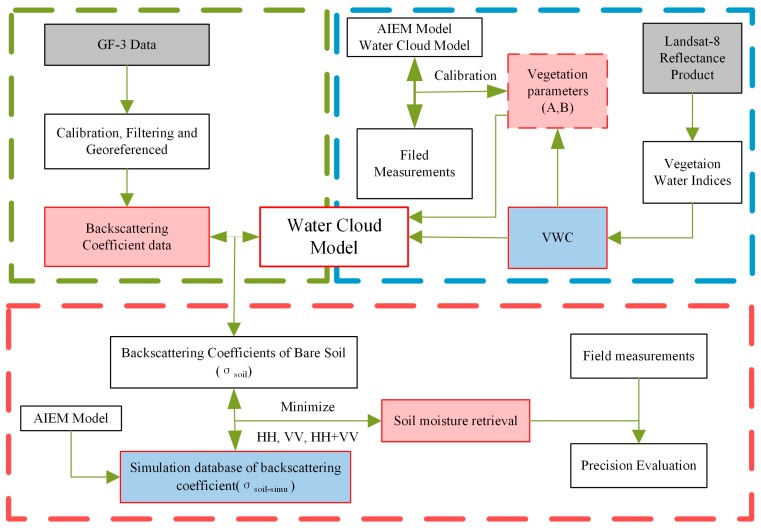
Flowchart of the proposed soil moisture retrieval algorithm for GF-3. AIEM: advanced integral equation model, GF-3: Gaofen-3 satellite, VWC: vegetation water content.

A flowchart of the proposed soil moisture retrieval algorithm developed for GF-3 satellite is presented in Figure 2. VWC was firstly calculated based on the Landsat-8 reflectance product and vegetation indices. A bare soil backscattering coefficient database was built through AIEM. Backscattering coefficient data from GF-3 data, vegetation parameters A and B, and VWC were inputted into the WCM to correct the effects of vegetation and obtain the backscattering coefficient of bare soil ($\sigma_{soil}^{{}^{\circ}}$). Then the values of $\sigma_{soil}^{{}^{\circ}}$ and the simulated bare soil backscattering coefficient $\sigma_{\text{soil-simu}}^{{}^{\circ}}$ were matched to search the closest data at the HH, VV, and HH+VV polarizations, respectively to retrieve soil moisture values. Finally, field soil moisture measurements were used to validate the accuracy of the estimated soil moisture.

### 4.1. Vegetation Water Indices

VWC (kg m^−2^) is one of the most important parameters used to estimate soil moisture from microwave remote sensing observations [40]. Based on moisture absorption peaks and valleys at 970, 1200, 1500, and 2200 nm, vegetation water indices can reflect changes in the water content of leaves, which can be calculated through Landsat-8 OLI reflectance data. At the pixel scale, the moisture—sensitive band is affected by the water content of single leaves and related to the number of leaves per unit volume [41]. Therefore, the vegetation water index calculated based on the moisture—sensitive band can reflect the water content in the vegetation canopy. Because various vegetation water indices can effectively reduce the scattering effects of single-band optical data, they have been widely used in VWC estimations. In this study, 8 vegetation water indices were investigated to establish their relationships with ground-based VWC measurements. These 8 vegetation water indices and their calculation formulas are shown in Table 2.

### 4.2. Bare Soil Backscatter Modeling by AIEM

The AIEM is a well-established theoretical model that seamlessly bridges the gap between small perturbation method (SPM) and Kirchhoff approximation (KA) models [12]. A comprehensive analysis and examination of the validity of AIEM was recently conducted, using extensive numerical and experimental data [13]. The results showed that AIEM accurately predicts scattering coefficients (for backscattering and bistatic scattering) and microwave emissivity under a wide range of geometric parameters and ground surface conditions [13]. Therefore, the AIEM model was used in this study to generate a bare soil backscatter database under the GF-3 configuration. A dielectric mixed model (i.e., the Dobson model [49]) was adopted to relate soil permittivity to soil moisture. The AIEM can be conceptually described as follows:

(1) $$\sigma_{\text{soil-simu}}^{{}^{\circ}}=AIEM(f,\theta ,PP,SM,s,l,ACF)$$

where *f* is the satellite frequency, *θ* is the incidence angle, *PP* is the polarization state, *SM* is the input soil moisture from the Dobson model, *s* is the root mean square height, *l* is the correlation length, and *ACF* is the adopted exponential autocorrelation function.

In the bare soil backscatter simulation database, *f* was the GF-3 satellite frequency (5.4 GHz), *θ* was set to a wide range (20–60 degrees with an interval of 1 degree. *PP* included HH and VV polarization, *s* and *l* were set based on in-situ measurements with 0.5–2.0 cm and 10.0–30.0 cm, respectively, with an interval of 0.1 cm and 1.0 cm, respectively. *SM* was set at 0.01–0.40 m^3^m^−3^ with an interval of 0.01 m^3^m^−3^, to cover the soil moisture range in most farmland areas.

### 4.3. Vegetation Backscatter Modeling by WCM

The Soil-vegetation scattering contribution can be simulated using physical vegetation scattering models [50,51]. However, these physical models require a detailed description of the vegetation morphology, which is typically not available at regional scales [52]. Therefore, soil moisture retrieval over agriculture fields is often based on the semi-empirical WCM, which assumes vegetation as a homogenous scatter [24]. In this study, WCM was used to simulate the total backscatter in vegetated areas. The WCM is described as follows:

(2) $$\sigma_{total}^{\circ }=\sigma_{veg}^{\circ }+\sigma_{veg+soil}^{\circ }+\tau^{2}\sigma_{soil}^{\circ }$$

where $\sigma_{total}^{{}^{\circ}}$ is the radar backscattering from the canopy, $\sigma_{veg}^{{}^{\circ}}$ is the vegetation scattering contribution (m^2^m^−2^),$\sigma_{soil}^{{}^{\circ}}$ is the soil surface scattering (m^2^m^−2^) and $\tau^{2}$ is the two-way attenuation. Interactions between vegetation and soil $\sigma_{veg+soil}^{{}^{\circ}}$ are neglected in the water-cloud model for its low proportion in total backscatter [25,26,52,53], and therefore, the WCM can be reformulated as follows:

(3) $$\sigma_{total}^{\circ }=\sigma_{veg}^{{}^{\circ}}+\tau^{2}\sigma_{soil}^{{}^{\circ}}$$

The WCM approximates $\sigma_{veg}^{{}^{\circ}}$ and $\tau^{2}$ as:

(4) $$\sigma_{veg}^{{}^{\circ}}=(1-\tau^{2})A\cdot m_{veg}\cos\theta \;$$

(5) $$\tau^{2}=\exp\left[\frac{-2B\cdot m_{veg}}{\cos\theta }\right]$$

where *θ* is the incidence angle, and *m_veg_* is the VWC (kg m^−2^), which is described in Section 4.1. *A* and *B* are both polarization dependent and crop dependent parameters, which can be calculated using the least squares method combined with simulated soil surface scattering, GF-3 measured backscattering and VWC.

### 4.4. Soil Moisture Retrieval Method 

*Step 1*: Calculation of VWC based on the optical vegetation index. Vegetation optical depth which is usually characterized by VWC, is the main factor affecting the contribution of vegetation to the reflected radar signal in the WCM. Therefore, 8 optical vegetation indices (see Section 4.1) were adopted to determine their relationships with the VWC by the combined use of Landsat-8 data and field measurements. Finally, the index that best fit the measured VWC among the 8 indices was adopted to correct the effects of vegetation optical depth.

*Step 2*: Generation of bare soil backscatter simulation database under the GF-3 configuration. Combined with ground-based measurement data and GF-3 satellite parameters, the AIEM was used to simulate the bare soil backscatter ($\sigma_{\text{soil-simu}}^{{}^{\circ}}$) of HH and VV polarization and their corresponding soil moisture and surface roughness parameters. During this process, the Dobson dielectric mixed model was adopted to relate soil permittivity to soil moisture. The model and parameter settings are introduced and detailed in Section 4.2.

*Step 3*: Calculation of bare soil backscatter ($\sigma_{soil}^{{}^{\circ}}$) using the WCM. The objective of this step is to correct the effects of vegetation on the backscattering coefficients and obtain the bare soil backscatter ($\sigma_{soil}^{{}^{\circ}}$). *m_veg_* is a parameter of the WCM, and was calculated in Step 1. The simulated bare soil backscatter ($\sigma_{\text{soil-simu}}^{{}^{\circ}}$) and *m_veg_* were inputted into the WCM to calculate parameters A and B using the least squares method. Finally, bare soil backscatter ($\sigma_{soil}^{{}^{\circ}}$) can be calculated based on the WCM. 

**Figure 3:**
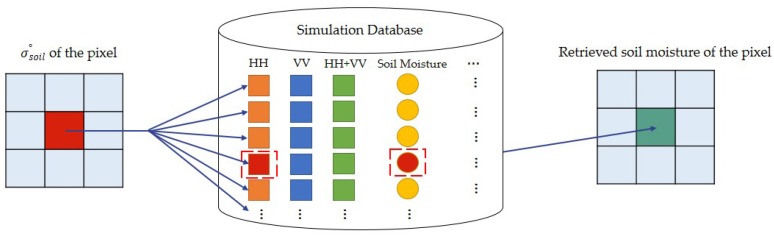
Soil moisture retrieval process based on the simulation database, using HH polarization as an example.

*Step 4*: Soil moisture retrieval based on the simulation database. The differences in simulated bare soil backscatter ($\sigma_{\text{soil-simu}}^{{}^{\circ}}$) and observed bare soil backscatter ($\sigma_{soil}^{{}^{\circ}}$) were minimized by cost function Z to obtain the soil moisture. The detailed process is shown in Figure 3, taking HH polarization as an example. To compare the HH, VV, and HH+VV precision, ground measurement data were used to validate the soil moisture values estimated from GF-3 data using the proposed algorithm with two statistical indices: the correlation coefficient R and root mean square error (RMSE). 

The cost function for HH and VV:

(6) $$Z=\min\sqrt{\frac{1}{n}\sum {\left(\sigma_{soil}^{{}^{\circ}}-\sigma_{\text{soil-simu}}^{{}^{\circ}}\right)}^{2}}$$

and for HH+VV:

(7) $$Z=\min\sqrt{\frac{1}{n}\sum {\left(|\sigma_{soil}^{{}^{\circ}}-\sigma_{\text{soil-simu}}^{{}^{\circ}}{|}_{HH}+|\sigma_{soil}^{{}^{\circ}}-\sigma_{\text{soil-simu}}^{{}^{\circ}}{|}_{VV}\right)}^{2}}$$

## 5. Results and Discussion

### 5.1. Vegetation Water Content 

**Figure 4:**
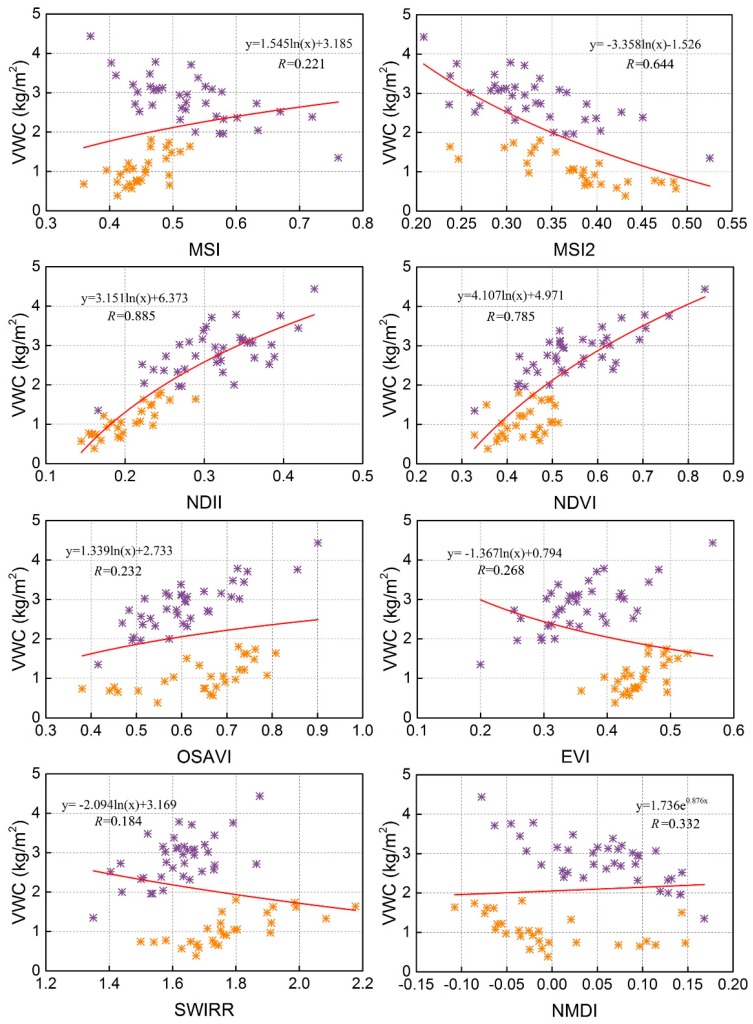
Relationships between different vegetation water indices from Landsat-8 data and measured VWC (the purple sampling points are wheat and the orange sampling points are corn).

VWC is an important input parameter in the WCM and should be derived before the WCM is implemented. Field measurements of VWC including wheat and corn were mixed and used simultaneously to analyze the applicability of eight vegetation water indices (Table 2) to determine the optimal index to use in the algorithm. Field VWC value of wheat (the purple sampling points) are higher than corn (the orange sampling points) for the wheat belongs to the filling stage and corn is in jointing stage. Figure 4 shows the relationship between these indices and VWC. Most indices had a logarithmic relationship with VWC. Only the normalized multi-band drought index (NMDI) exhibited an exponential relationship that included negative values. Normalized difference infrared index (NDII), Normalized difference vegetation index (NDVI) and Moisture stress index 2 (MSI2) have a better relationship with VWC both for the crop of wheat and corn. The remaining five indices exhibit weaker relationships with mixed VWC of wheat and corn with respect to the separate relationship with wheat or corn. 

To analyze the ability of various vegetation water indices in estimating VWC, we calculated *R* and RMSE values for the vegetation water indices (Table 3). To obtain a better fit between the vegetation water indices and VWC, *R* and RMSE values should be closer to 1.0 and 0.0, respectively. Our results showed that the eight indices have varying applicability for both wheat and corn with *R* ranging from 0.184 to 0.885. The regression line for each index fit the field VWC values at different levels of precision. NDII, NDVI and MSI2 had higher precision (*R* ≥ 0.644, RMSE ≤ 0.795 kg m^−2^) than the remaining vegetation water indices(*R* ≤ 0.332, RMSE ≥ 1.001 kg m^−2^). Among the eight indices, NDII exhibited the most precise fit (*R* = 0.885, RMSE = 0.484 kg m^−2^) and had the best applicability for both wheat and corn. Therefore, NDII was selected to retrieve VWC in this study with the following regression equation:

(8) VWC=3.151ln(NDII)+6.373

**Figure 5:**
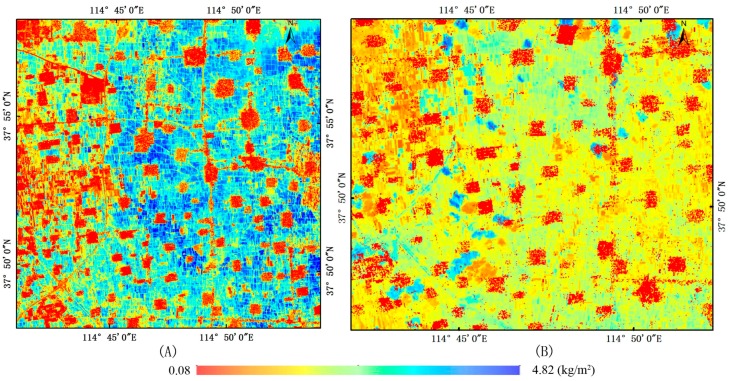
VWC derived from Landsat-8 OLI data (kg m^−2^) ((**A**) wheat in the filling stage, (**B**) corn in jointing stage).

The *VWC* results calculated by equation 8 based on Landsat-8 OLI data are shown in Figure 5. Figure 5A is the VWC results of crop of wheat in the filling stage and Figure 5B is corn in jointing stage. VWC results are generally different due to different crop type, planting dates and growth statuses. This phenomenon is well represented in Figure 5, manifested by the block distribution structure of VWC. Dark blue patches represent higher VWC in farmland, followed by light blue and yellow patches. Red patches mainly represent facilities such as township residential areas and roads. Through the VWC analysis, we found the difference in VWC between different plots was very large, approaching 4 kg m^−^^2^. VWC of wheat in the filling stage are higher than corn in jointing stage. The VWC results of wheat are mainly shown by blue patches, and the corn are mainly shown by yellow patches (the area of blue patches is the cloud cover area).

### 5.2. Bare Soil Backscattering 

**Figure 6:**
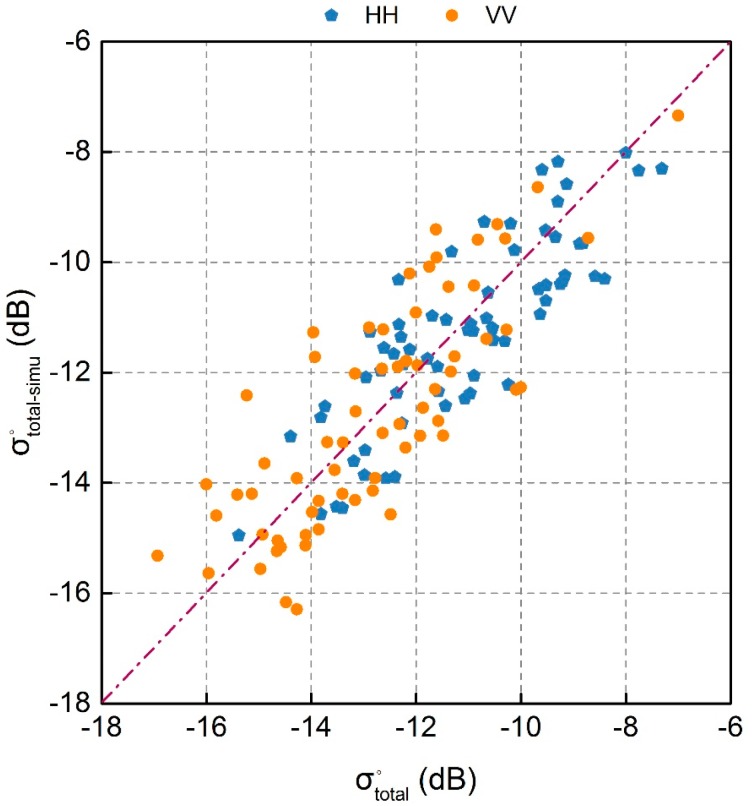
Scatterplot of simulated radar backscatter ($\sigma_{\text{soil-simu}}^{{}^{\circ}}$) from WCM and GF-3 measured radar backscatter ($\sigma_{total}^{{}^{\circ}}$).

The parameters of A and B in WCM were calculated based on the method mentioned in Section 4.3. And then the total radar backscatter ($\sigma_{\text{soil-simu}}^{{}^{\circ}}$) were simulated based on WCM combined with bare soil backscatter ($\sigma_{\text{soil-simu}}^{{}^{\circ}}$) simulated by AIEM and VWC calculated from NDII. In order to evaluate the validity of WCM for vegetation backscatter modelling under GF-3 satellite configuration, the simulated radar backscatter ($\sigma_{\text{soil-simu}}^{{}^{\circ}}$) were compared with the GF-3 measured radar backscatter ($\sigma_{total}^{{}^{\circ}}$), shown in Figure 6. Both HH and VV simulated backscatter agree well with GF-3 satellite measured backscatter (R = 0.839 for HH, and R = 0.797 for VV). The results also indicate the calculated parameters of A and B were suitable for the study area. Therefore, WCM was chosen to correct the effects of vegetation.

**Figure 7:**
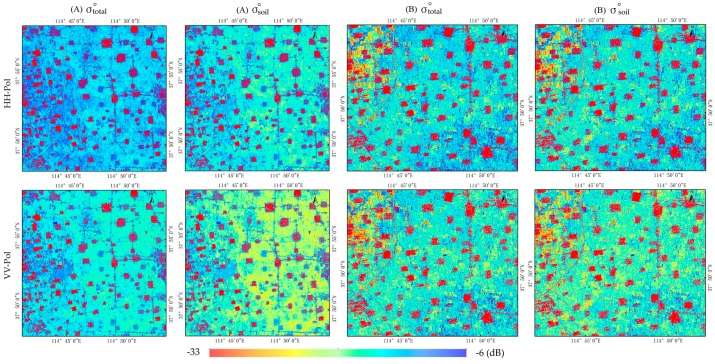
GF-3 satellite backscattering coefficient image ($\sigma_{total}^{{}^{\circ}}$) before and ($\sigma_{soil}^{{}^{\circ}}$) after implementing the water-cloud model ((**A**) wheat in the filling stage, (**B**) corn in jointing stage).

The total radar backscatter from the canopy ($\sigma_{total}^{{}^{\circ}}$) and the bare soil backscatter ($\sigma_{soil}^{{}^{\circ}}$) extracted based on the WCM by correcting the effects of vegetation are shown in Figure 7. Backscattering coefficient values were clearly attenuated after removing the contribution of vegetation in agricultural fields (patches changed from blue to yellow). This indicates the effectiveness of the WCM in eliminating canopy backscattering, which is consistent with previous results [26,28]. During the soil moisture retrieval process, the backscattering coefficient will be used to match soil surface scattering ($\sigma_{\text{soil-simu}}^{{}^{\circ}}$) simulated by AIEM. 

**Figure 8:**
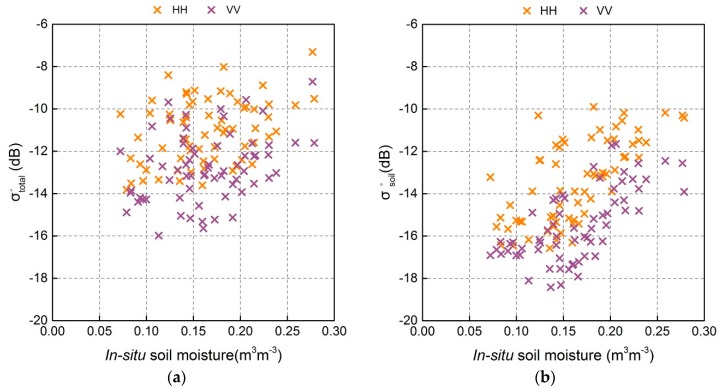
Correlations between in-situ soil moisture and (**a**) total backscatter, (**b**) bare soil backscatter.

We also investigated the responses of the HH-pol, and VV-pol backscatter of $\sigma_{total}^{{}^{\circ}}$ and $\sigma_{soil}^{{}^{\circ}}$ to in-situ soil moisture to explore whether the correlation between the backscattering coefficient and soil moisture had improved following the elimination of the contribution of vegetation, shown in Figure 8. It is observed that all radar observations generally exhibit correlation with soil moisture, which is consistent with previous findings [8], demonstrating the good potential of GF-3 observations for estimating soil moisture. As expected, $\sigma_{soil}^{{}^{\circ}}$ backscatter correlated better with soil moisture than $\sigma_{total}^{{}^{\circ}}$ for HH-pol and VV-pol (*R* > 0.650, Table 4). The *R* value for HH-pol $\sigma_{soil}^{{}^{\circ}}$ data was 0.665, slightly higher than that of VV-pol data (0.651). The dynamic range of $\sigma_{soil}^{{}^{\circ}}$ was also greater than that of $\sigma_{total}^{{}^{\circ}}$. These results demonstrate the importance and necessity to eliminate the effects of vegetation in soil moisture retrieval. The scattering mechanism between backscatter and soil moisture is very complex. In addition to soil moisture, other soil parameters such as surface roughness affect radar backscatter. If we assume a simple linear or nonlinear fitting relationship between $\sigma_{soil}^{{}^{\circ}}$ and soil moisture, the *R*^2^ value becomes lower than 0.430 (for HH, R = 0.665, for VV, R = 0.651); i.e., soil moisture retrievals would be performed with insufficient precision. AIEM is a well-established physical model that comprehensively considers the influence of surface parameters on backscatter. Therefore, AIEM was used to carry out the soil moisture inversion in this study; the results are described in Section 5.3.

### 5.3. Evaluation of Soil Moisture Retrievals

**Figure 9:**
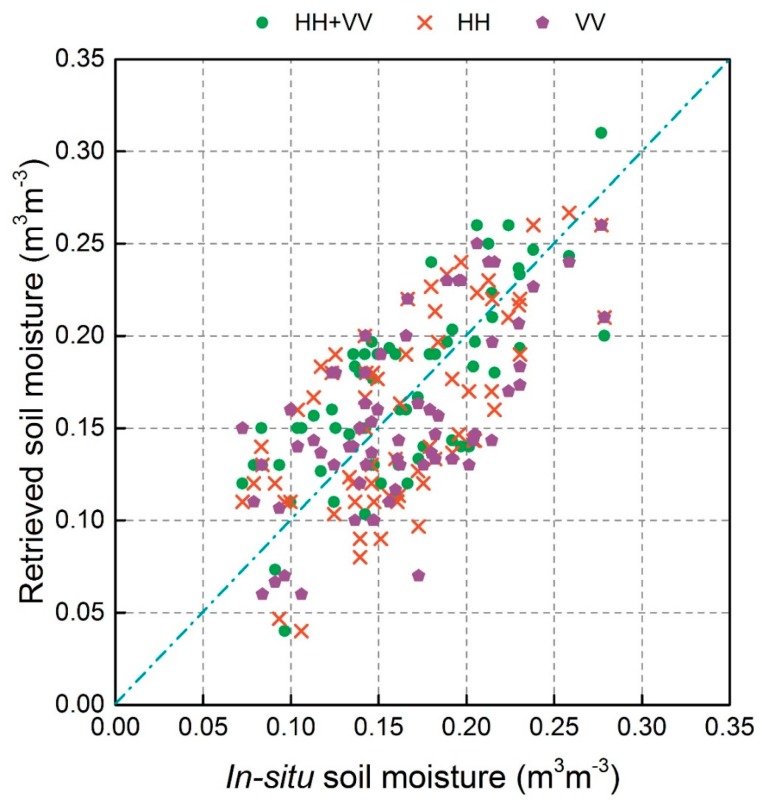
Scatterplots of in-situ soil moisture and soil moisture retrieved from the HH+VV, HH, and VV Polarizations.

**Figure 10:**
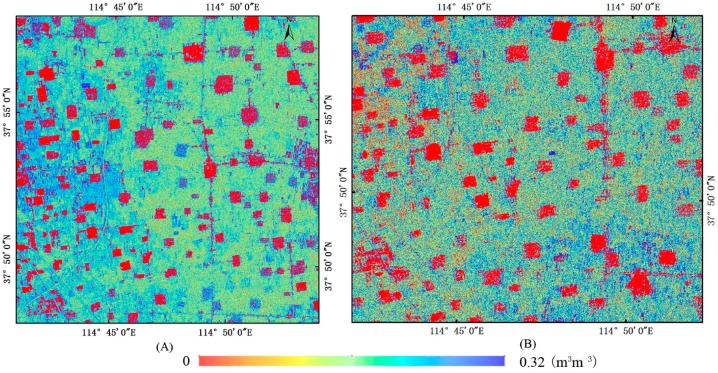
Retrieved soil moisture content map from the GF-3 satellite based on HH+VV backscatter ((**A**) wheat in the filling stage, (**B**) corn in jointing stage).

A bare soil backscatter simulation database was built using the AIEM model; this database included $\sigma_{\text{soil-simu}}^{{}^{\circ}}$ backscatter for HH and VV polarization and their corresponding soil moisture and surface roughness parameters. The soil moisture retrievals were obtained by minimizing the differences between the simulated bare backscattering coefficients ($\sigma_{\text{soil-simu}}^{{}^{\circ}}$) and observed bare soil backscatters ($\sigma_{soil}^{{}^{\circ}}$). To compare the precision of the three ways (HH, VV, and HH+VV), 64 field measured data were used to validate the soil moisture estimated from GF-3 data using the proposed algorithm, shown in Figure 9. HH, VV, and HH+VV displayed good performance in estimating soil moisture with correlation coefficients greater than 0.636, shown in Table 5. The HH+VV combination showed the best precision (RMSE = 0.044 m^3^m^−3^), followed by HH (0.049 m^3^m^−3^) and VV (0.053 m^3^m^−3^). Therefore, both HH and VV polarized backscatter from the GF-3 satellite were chosen to retrieve soil moisture for the entire study area, shown in Figure 10. The spatial distribution of soil moisture content was visually reasonable. Because some farmlands were irrigated separately, moisture levels were higher in these farmlands than surrounding areas. Therefore, aside from the precision evaluation by in-situ data, visual observation of spatial characteristics of soil moisture estimated using GF-3 satellite data can preliminarily indicate the reasonability of the proposed soil moisture retrieval algorithm.

## 6. Conclusions

The GF-3 satellite, launched on 10 August 2016, is the first Chinese C-band multi-polarization civil SAR which has large coverage and fine scale by 12 imaging modes. To explore its capability for estimating soil moisture, we developed a soil moisture retrieval algorithm for agricultural fields using GF-3 data based on the WCM and a simulated backscattering coefficient database built using AIEM. Ground-based measurements at wheat and corn areas were used to evaluate the accuracy of the proposed algorithm. The results indicated that the difference between in-situ and retrieved soil moisture was small and that the soil moisture results were satisfactory (*R* > 0.636, RMSE ≤ 0.053 m^3^m^−3^ for HH, VV, and HH+VV polarization). The results also demonstrated the reliability of the proposed soil moisture retrieval algorithm developed for GF-3 satellite and that GF-3 data could yield good soil moisture estimation results. The major conclusions of this study are summarized as follows.

VWC has a great impact on the accuracy of soil moisture estimation over agricultural fields. Based on eight vegetation water indices, we selected the most suitable index for VWC estimation by determining the relationships between these indices and field VWC. NDII was used to calculate VWC with the highest accuracy (*R* = 0.885, RMSE = 0.484 kg m^−2^). VWC varies spatially due to different crop types, planting dates and growth statuses, and this phenomenon was well reflected by the use of NDII. The difference between the maximum and minimum VWC can reach as high as 4 kg m^−2^, which indicates that accurate VWC correction is crucial; errors in VWC calculation directly influence the precision of soil moisture estimations. 

The WCM was used to eliminate the scattering contribution from vegetation to extract bare soil backscattering ($\sigma_{soil}^{{}^{\circ}}$). The responses of HH-pol and VV-pol backscatter of $\sigma_{total}^{{}^{\circ}}$ and $\sigma_{soil}^{{}^{\circ}}$ backscatter to in-situ soil moisture were also investigated. The results showed that $\sigma_{soil}^{{}^{\circ}}$ correlated better with soil moisture than $\sigma_{total}^{{}^{\circ}}$ both at HH-pol and VV-pol (*R* > 0.650), demonstrating the necessity of correcting the effects of vegetation for reliable soil moisture estimations. 

A simulation database including $\sigma_{\text{soil-simu}}^{{}^{\circ}}$ backscatters, covering a wide range of surface roughness and soil moisture content, was built based on AIEM. Soil moisture estimation was achieved by minimizing $\sigma_{soil}^{{}^{\circ}}$ and $\sigma_{\text{soil-simu}}^{{}^{\circ}}$. Ground-based soil moisture data were used to evaluate the accuracy of estimates obtained from GF-3 data using HH, VV and HH+VV polarizations respectively. All three schemes showed highly accurate soil moisture retrievals (*R* > 0.636). Among them, the HH+VV scheme exhibited the highest precision, with the lowest RMSE value (0.044 m^3^m^−3^).

Although the proposed soil moisture algorithm achieved satisfactory soil moisture retrievals using GF-3 satellite data, there were some limitations need to be noted. GF-3 radar operates in quad-polarization imaging mode, while we analyzed the performance of soil moisture retrieval using co-polarization only. This is because the contribution of multiple scattering, which is dominant in cross- polarization for backscattering, is not considered in the current AIEM model [13]. Meanwhile, due to the lack of simulated soil surface scattering of cross-polarization, WCM is not used to calculate the cross-pol total radar backscatter in our study. Therefore, we will add cross-polarized backscatter (HV or VH) values in the future to further improve the accuracy of soil moisture retrievals by including multiple scattering effects in the AIEM model. The vegetation parameter A and B of WCM are both polarization and crop dependent parameters, which need to be obtained combining with field measurements data of the research area. Therefore, obtaining parameter A and B applied to the entire globe is also the focus of our future research. Lastly, soil moisture retrieval algorithm proposed at this study was validated in an agriculture region with wheat and corn as the dominant crop type due to limited GF-3 data available for us. In future study, we will validate the proposed algorithm in more regions representing different surface conditions. 

## Figures and Tables

**Table 1:** Main technical specifications of QPSI imaging mode of the GF-3 satellite.

Imaging Mode	Look Number	Resolution (m)			Imaging Bandwidth (km)		Polarization Mode
		Nominal	Azimuth	Range	Nominal	Size	
Quad-polarization strip I	1 × 1	8	8	6~9	30	20~35	Full polarization

**Table 2:** Formulas used to calculate the 8 vegetation water indices in this study.

Vegetation Water Indices	Formula	Reference
Moisture stress index (MSI)	$\mathrm{MSI}=\frac{\rho_{swir1}}{\rho_{nir}}$	[42]
Moisture stress index 2 (MSI2)	$\mathrm{MSI}2=\frac{\rho_{swir2}}{\rho_{nir}}$	[43]
Normalized multi-band drought index (NMDI)	$\mathrm{NMDI}=\frac{\rho_{red}-\left(\rho_{swir1}-\rho_{swir2}\right)}{\rho_{red}+\left(\rho_{swir1}+\rho_{swir2}\right)}$	[44]
Normalized difference infrared index (NDII)	$\mathrm{NDII}=\frac{\rho_{nir}-\rho_{swir1}}{\rho_{nir}+\rho_{swir1}}$	[45]
Normalized difference vegetation index (NDVI)	$\mathrm{NDVI}=\frac{\rho_{nir}-\rho_{red}}{\rho_{nir}+\rho_{red}}$	[46]
Enhanced vegetation index (EVI)	$\mathrm{EVI}=2.5*\frac{\rho_{nir}-\rho_{red}}{\rho_{nir}+6*\rho_{red}-7.5*\rho_{blue}+1}$	[47]
Shortwave infrared ratio (SWIRR)	$\mathrm{SWIRR}=\frac{\rho_{swir1}}{\rho_{swir2}}$	[43]
Optimized soil adjusted vegetation index (OSAVI)	$\mathrm{OSAVI}=\frac{\left(1+0.16\right)*\left(\rho_{nir}-\rho_{red}\right)}{\rho_{nir}+\rho_{red}+0.16}$	[48]

For the Landsat 8 OLI, $\rho_{red}$ is the Band 4 Red, $\rho_{blue}$ is the Band 2 Blue, $\rho_{nir}$ is the Band 5 NIR, $\rho_{swir1}$ is the Band 6 Swir 1, $\rho_{swir2}$ is the Band 7 Swir 2.

**Table 3:** Error metrics of vegetation water content (VWC) derived from different vegetation water indices.

Vegetation Water Indices	*R*	RMSE (kg m^−2^)
MSI	0.221	1.014
MSI2	0.644	0.795
NMDI	0.332	1.037
NDII	0.885	0.484
NDVI	0.785	0.644
EVI	0.268	1.001
SWIRR	0.184	1.022
OSAVI	0.232	1.011

*R*: correlation coefficient, RMSE: root mean square error.

**Table 4:** Correlation of $\sigma_{total}^{{}^{\circ}}$ and $\sigma_{soil}^{{}^{\circ}}$ backscatter with in-situ soil moisture.

Polarization	$\sigma_{total}^{{}^{\circ}}$	$\sigma_{soil}^{{}^{\circ}}$
HH	0.383	0.665
VV	0.327	0.651

**Table 5:** Error metrics of soil moisture data retrieved from different polarizations.

Polarization	*R*	RMSE (m^3^m^−3^)
HH	0.652	0.049
VV	0.636	0.053
HH+VV	0.717	0.044
